# *Actinopyga lecanora* Hydrolysates as Natural Antibacterial Agents

**DOI:** 10.3390/ijms131216796

**Published:** 2012-12-07

**Authors:** Raheleh Ghanbari, Afshin Ebrahimpour, Azizah Abdul-Hamid, Amin Ismail, Nazamid Saari

**Affiliations:** 1 Faculty of Food Science and Technology, University Putra Malaysia, 43400 UPM Serdang, Selangor, Malaysia; E-Mails: raheleghanbari@yahoo.com (R.G.); a_ebrahimpour@yahoo.com (A.E.); azizah@food.upm.edu.my (A.A.-H.); 2 Faculty of Medicine and Health Sciences, University Putra Malaysia, 43400 UPM Serdang, Selangor, Malaysia; E-Mail: amin@medic.upm.edu.my

**Keywords:** *Actinopyga lecanora*, bioactive peptides, antibacterial activity, proteolytic enzyme

## Abstract

*Actinopyga lecanora*, a type of sea cucumber commonly known as stone fish with relatively high protein content, was explored as raw material for bioactive peptides production. Six proteolytic enzymes, namely alcalase, papain, pepsin, trypsin, bromelain and flavourzyme were used to hydrolyze *A. lecanora* at different times and their respective degrees of hydrolysis (DH) were calculated. Subsequently, antibacterial activity of the *A. lecanora* hydrolysates, against some common pathogenic Gram positive bacteria (*Bacillus subtilis* and *Staphylococcus aureus*) and Gram negative bacteria (*Escherichia coli*, *Pseudomonas aeruginosa*, and *Pseudomonas* sp.) were evaluated. Papain hydrolysis showed the highest DH value (89.44%), followed by alcalase hydrolysis (83.35%). Bromelain hydrolysate after one and seven hours of hydrolysis exhibited the highest antibacterial activities against *Pseudomonas* sp., *P. aeruginosa* and *E. coli* at 51.85%, 30.07% and 30.45%, respectively compared to the other hydrolysates. Protein hydrolysate generated by papain after 8 h hydrolysis showed maximum antibacterial activity against *S. aureus* at 20.19%. The potent hydrolysates were further fractionated using RP-HPLC and antibacterial activity of the collected fractions from each hydrolysate were evaluated, wherein among them only three fractions from the bromelain hydrolysates exhibited inhibitory activities against *Pseudomonas* sp., *P. aeruginosa* and *E. coli* at 24%, 25.5% and 27.1%, respectively and one fraction of papain hydrolysate showed antibacterial activity of 33.1% against *S. aureus.* The evaluation of the relationship between DH and antibacterial activities of papain and bromelain hydrolysates revealed a meaningful correlation of four and six order functions.

## 1. Introduction

The demands for natural antimicrobial compounds that are effective and non-toxic with less environmental risk has greatly increased due to the rising bacterial resistance against the synthetic antimicrobial agents and limitation on the use of synthetic preservatives in food systems. Therefore, exploring new food grade antimicrobial compounds from natural sources is a tall order. Recently, bioactive peptides with antibacterial activity have received a great attention in food industry, due to their low toxicity and unique biological mechanisms of disrupting the membrane of the pathogens [1].

Apart from antimicrobial activity, bioactive peptides are also known to exhibit other functional properties such as antihypertensive, immunomodulating, anti-thrombotic, antioxidative, anticancer, and antithrombotic [2]. These bioactive peptides are liberated through hydrolysis from the parent protein structure where they are inactive [3,4]. Hydrolysates of antimicrobial peptides (AMPs) are of interest to many researchers as they can be used as a potential source of natural preservatives. AMPs found in nature basically varied in length, structure and amino acid composition [5] with less than 100 amino acid residues [6]. Most of them are linear with a potential to form amphipathic α-helical or β-sheet structures. In spite of these variations, they kill bacteria through similar mechanisms such as membrane disruption, metabolism interference and interacting with intracellular compounds [6].

Several antimicrobial bioactive peptides of marine invertebrates, including American lobster [7] oyster [8], shrimp [9] and green sea urchin [10] have been produced through enzymatic hydrolysis. A novel cysteine-rich antimicrobial peptide, CgPep33, that exhibiting activity against bacteria such as *E. coli*, *B. subtilis*, *P. aeruginosa* and fungi was isolated from oyster (*Crassostrea gigas*) proteolysate [8]. Mingyong *et al.* (2008) [11] generated a peptide fraction (molecular weight 5–10 kDa) from oyster (*Crassostrea gigas*) by alcalase and bromelin treatment that exhibited an inhibitory activity against the herpes virus [11]. Battison *et al.* (2008) [7] produced and partially characterised two antimicrobial peptides from haemocytes of the American lobster. These antimicrobial peptides exhibited bacteriostatic activity against some Gram negative bacteria and both protozoastatic and protozoacidal activities.

Sea cucumber is widely used as a human food source in Asian countries such as Philippines, China, Japan, Korea and Malaysia [5]. Nutritionally, it is a valuable food source due to its high protein content and low level of fat [12]. It has been used in East Asian countries as the traditional remedy to treat wounds, eczema, arthritis and hypertension. Collagen and eicosapentaenoic acid (EPA) of sea cucumber are valuable nutrient supplements, which promote the formation of blood cells (hematogenesis) [13], tissue repairing and wound healing [14]. A number of studies have been conducted to characterize and determine the biological and medicinal activities of different sea cucumber species [15]. The antioxidant, antimicrobial, antifungal, antinociceptive and wound healing properties of some sea cucumbers have been reported [14,16–18]. These pharmacological properties are related to the presence of different bioactive compounds such as triterpene glycosides (saponins) [19–21], chondroitin sulfates [22], glycosaminoglycan [23,24], sulphated polysaccharides [25], sterols (glycosides and sulfates) [26], phenolics [27], peptides [28], cerberosides [29] and lectins [30,31]. *Actinopyga lecanora*, commonly known as stone fish is classified among edible species of sea cucumber [32]. *A. lecanora* belongs to the marine invertebrate of the phylum Echinoderm and the Holothuroidea class, a by-catch of fishery industry, which it is not usually consumed as food in Malaysia. Hence, due to its relatively high protein content, *A. lecanora* would be a potential commercial source for bioactive peptides generation. To the best of our knowledge, there is no wellestablished scientific data reported on the properties of bioactive peptides such as antibacterial activity derived from *A. lecanora*. Therefore, the main objective of the present study was to explore the antibacterial activity of peptides generated from *A. lecanora* by enzymatic hydrolysis.

## 2. Results and Discussion

### 2.1. Proximate Composition

The proximate composition of *A. lecanora* as raw material for hydrolysis was analyzed (Table 1). The protein content of *A. lecanora* (7.03 g/100 g), was close to that of other sea cucumber species, namely *Holothuria polii* (8.66 g/100 g), *Holothuria tubulosa* (8.82 g/100 g) and *Holothuria mammata* (7.88 g/100 g), but it was higher than the protein content of *Stichopus horrens* (2.83 g/100 g) [33]. Chang-Lee *et al.* (1989) [34] pointed out a wide range of proximate compositional data for fresh sea cucumbers (Table 1). The findings of the present study for moisture, protein, fat and ash contents were in agreement with the range reported.

### 2.2. Degree of Hydrolysis

**Figure 1:**
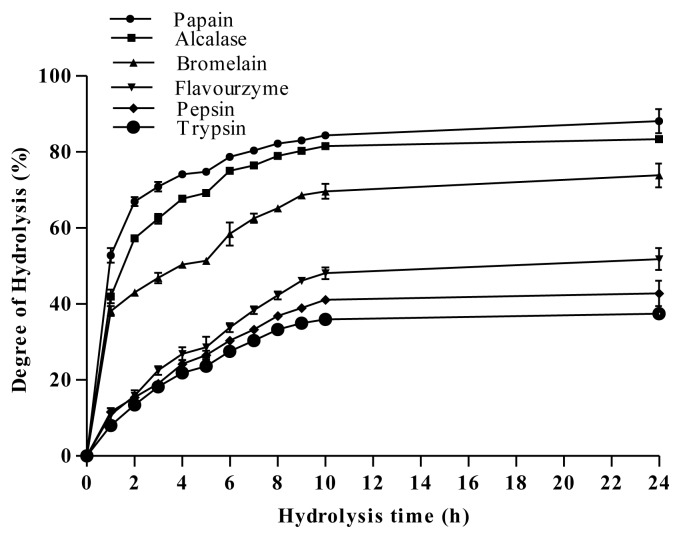
Degree of hydrolysis of *A. lecanora*, hydrolyzed by different proteolytic enzymes. Results are the average of triplicate determinations (mean ± SD).

In order to produce antibacterial peptides, *A. lecanora* was hydrolyzed using different classes of proteases namely cysteine proteases (bromelain and papain), serine proteases (trypsin and alcalase), aspartate protease (pepsin) and exopeptidase protease (flavourzyme) for 24 h. For production of peptides, the extent of protein hydrolyses with time was monitored by measuring the degree of hydrolysis (DH). DH has been defined as the percent ratio of the number of peptide bonds cleaved to the total number of peptide bonds within the substrate used [35]. It is the proportion of cleaved peptide bonds in a protein hydrolysate. Therefore, DH is the most widely used indicator for comparing different protein hydrolysates. During enzymatic hydrolysis, cleavage of peptide bonds releases the α-amino groups, which are reacted with OPA in the presence of β-mercaptoethanol forming a complex compound detectable at absorbance of 340 nm. Figure 1 depicts that the patterns of *A. lecanora* hydrolysis by different proteases were found to be similar, but with significant (*p* < 0.05) differences in DH values.

Three different phases are observed. The first phase showed an initial rapid-digestion for 1 h after the addition of enzyme, indicating that cleavage sites were available for enzyme to act on. In the second phase, the extent of hydrolysis steadily decreased until reaching a plateau (third phase). The plateau phase could either be due to the limitation of the available enzyme cleavage sites, enzyme inactivation and/or product inhibition. This plateau state remained for the next 14 h implying that the hydrolysis was completed.

Among the various enzymes tested, papain was the fastest and the most efficient enzyme for hydrolysis of *A. lecanora*, followed by alcalase, bromelain, flavourzyme, pepsin and trypsin with DH values of 89.44%, 83.35%, 73.84%, 51.80%, 42.77%, and 37.46%, respectively (Figure 1). Basically, the ability of enzyme to cleave peptide bonds depends on the enzyme/substrate ratio and accessibility of the enzyme to the substrate cleavage sites [36]. Furthermore, the difference in DH is a result of the difference in the total number of cleavage sites of the substrate. Therefore, it can be concluded that although *A. lecanora* was degradable by all six proteases, the number of available cleavage sites for papain was much higher than other proteases used. Being an endoprotesae, the efficiency of papain also is in line with its broad specificity towards a protein substrate.

### 2.3. Antibacterial Activity of Hydrolysates

**Figure 2:**
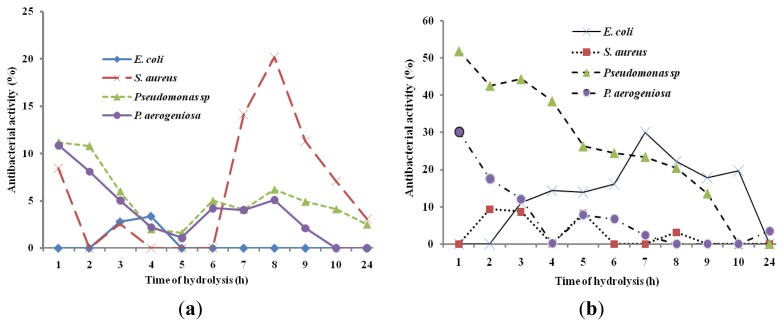
Bacterial growth inhibitions (%) of *A. Lecanora* hydrolysates produced by bromelain (**a**) and papain (**b**). Results are the average of triplicate determinations (mean ± SD).

In order to study the antibacterial activity of *A. lecanora* hydrolysates, samples were withdrawn every 1 h of hydrolysis for up to 24 h and assayed for antibacterial activities. The antibacterial abilities of different hydrolysates against *E. coli*, *B. subtilis*, *S. aureus*, *P. aeruginosa* and *Pseudomonas* sp. were studied. The hydrolysates produced by alcalase, flavourzyme, pepsin and trypsin did not show any antibacterial activities (data are not shown), while only hydrolysates produced by papain and bromelain exhibited antibacterial activities (Figure 2). In addition, the control (unhydrolyzed *A. lecanora*) exhibited no antibacterial effect.

The growth of *E. coli*, *P. aeruginosa* and *Pseudomonas* sp. was reduced by different bromelain hydrolysates by 30%, 30.07% and 51.85%, respectively (Figure 2a), whereas papain hydrolysate showed antibacterial activity of 20.19% against *S. aureus* (*p* < 0.05) (Figure 2b). Based on the results, hydrolysis time had a significant effect on the growth inhibition percentage of each bacterium (*p* < 0.05). Song *et al.* (2011) [37] revealed that protein hydrolysates of halffin anchovy displayed antibacterial activities against *E. coli*. In addition, Salampessy *et al.* (2010) [38] showed the antibacterial activity of leatherjacket (*Meuchenia* sp.) bromelain hydrolysates against *S. aureus* and *B. cereus.*

On the other hand, this finding showed that *A. lecanora* hydrolysates were more active in inhibiting the growth of Gram negative bacteria (*P. aeruginosa*, *Pseudomonas* sp., and *E. coli*) than that of Gram positive bacterium (*S. aureus*). Thus, the selections of suitable protease and time of hydrolysis are crucial due to the enzyme specificity and activity, producing peptides, which vary in molecular size, amino acid sequences and consequent differences in antibacterial activity.

Although bromelain is commonly used to enhance the hydrolysis or fermentation processes such as preparation of soy and fish sauces, there are a few reports showing the release of antibacterial peptides by this enzyme from different protein sources [38]. To the best of our knowledge, this may be the first finding of an antimicrobial peptide released through hydrolysis of *A. lecanora*. Thus, peptides derived from *A. lecanora* have the potential to be used as natural preservatives in food products as supported by the World Health Organization which emphasizes the use of natural preservatives in food [39]. Although bitterness is a usual characteristic of different protein hydrolysates, microencapsulation is a useful technique to mask or reduce the unpleasant flavor and hygroscopic property of the product, in addition to increasing its stability.

### 2.4. Relationship between Degree of Hydrolysis and Antibacterial Activity

**Figure 3:**
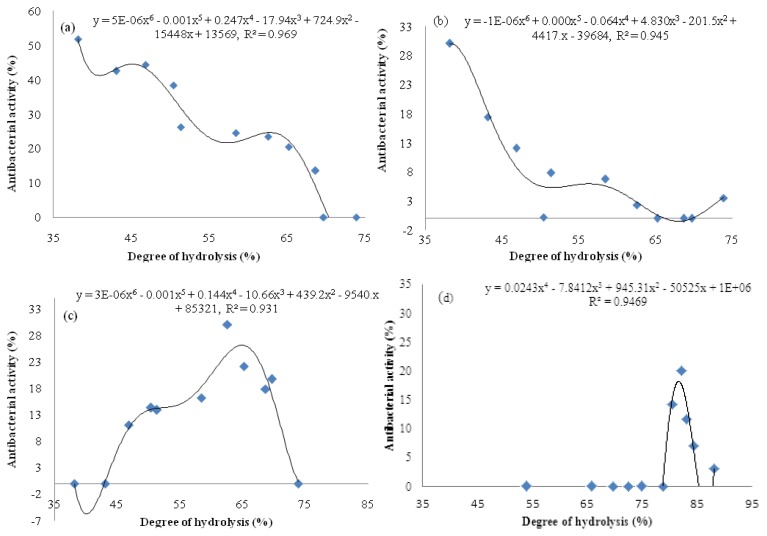
Relationship between DH and antibacterial activity of *A. lecanora* hydrolyzed by bromelain [(**a**) and (**b**)] and papain [(**c**) and (**d**)]: (**a**) *Pseudomonas* sp.; (**b**) *P. aerogeniosa*; (**c**) *E. coli*; and (**d**) *S. aureus*.

The degree of hydrolysis (DH) indicates the progress of hydrolysis for generating peptides of different sizes and amino acid sequences, where antibacterial activity depends on both peptides’ properties mentioned. Thus, it is crucial to determine any relationship that might exist between DH and antibacterial activity. Plotting DH *versus* antibacterial activity (Figure 3) showed that despite an increasing DH value during the hydrolysis, different antibacterial patterns were exhibited. The bromelain hydrolysis showed a downward trend antibacterial activity (Figure 3a,b) whereas papain hydrolysis exhibited a mixed pattern trend of antibacterial activity with two phases (Figure 3d): an increase in DH value up to 82% caused an increase in antibacterial activity value, while in second phase, antibacterial activity was decreased with the further increase in DH value. On the other hand, bromelain proteolysis showed a steadily decrease in antibacterial activity against *Pseudomonas* sp. and *P. aerogeniosa* with the DH increasing (Figure 3a,b). Correlation coefficient of more than 0.93 shows a meaningful relationship between DH and antibacterial activities of *A. lecanora* hydrolysates prepared to use bromelain and papain. The relationship followed a 4- and 6- order functions (Figure 3). On the other hand, since antibacterial activity of a peptide is affected by its amino acid sequence, secondary structure, length, molecular weight and charge, thus DH and antibacterial activity depend on the type of the protease used and the amino acid sequence of parent protein [7].

### 2.5. Antibacterial Activity of Fractionated Peptides

In this study, papain and bromelain generating active hydrolysates were further fractionated using reversed-phase HPLC (RP-HPLC) and the collected fractions were characterized for their antibacterial properties. In RP-HPLC, compounds are separated based on their hydrophobic characteristic where more hydrophobic peptides showed longer elution times in a RP column. In this process, the molecules are partitioned between C_18_ matrix and mobile phase, where the matrix is hydrophobic, and mobile phase consisting of a gradient mixture of solvents from relatively polar (hydrophilic) to relatively non polar (hydrophobic). Therefore, fractions (peptides) that come out in the early stage contain relatively more hydrophilic molecules and fractions come out in the later stage contain relatively more hydrophobic molecules.

**Figure 4:**
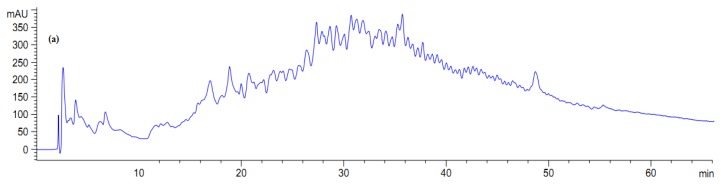

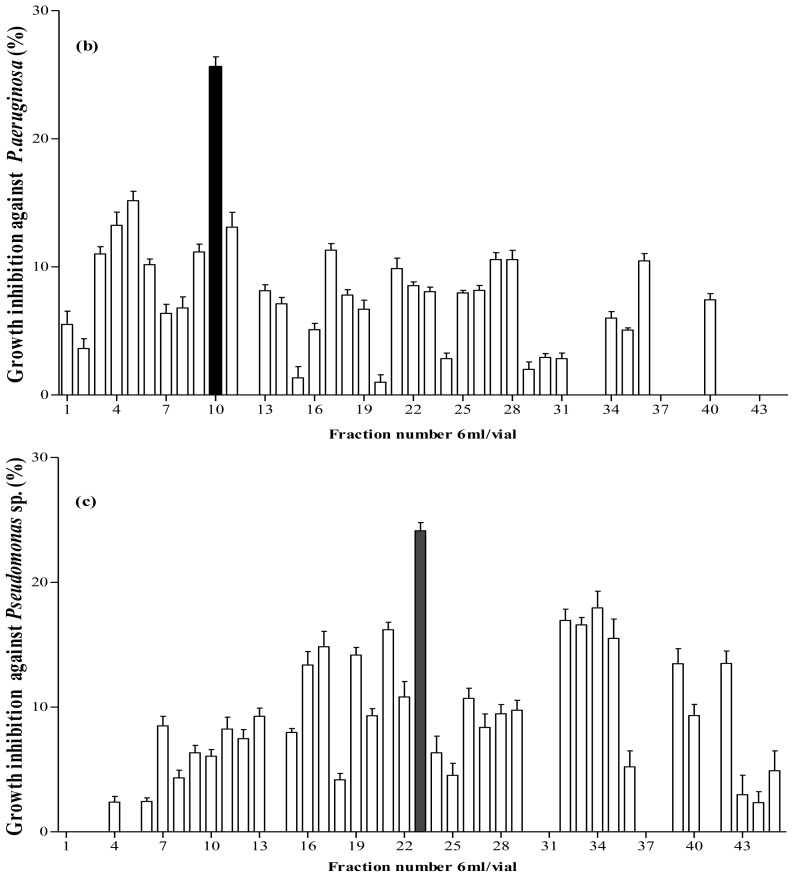
Fractionation of *A. lecanora* bromelain generated hydrolysate (after 1 h hydrolysis) by semi-preparative RP-HPLC. (**a**) Chromatogram of *A. lecanora* hydrolysate; (**b**) Growth inhibition (%) of collected fractions against *P. aerogeniosa*; and (**c**) Growth inhibition (%) of collected fractions against *Pseudomonas* sp.

**Figure 5:**
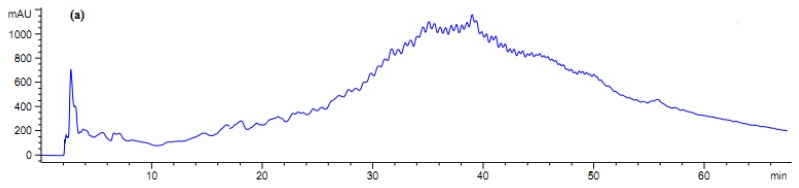

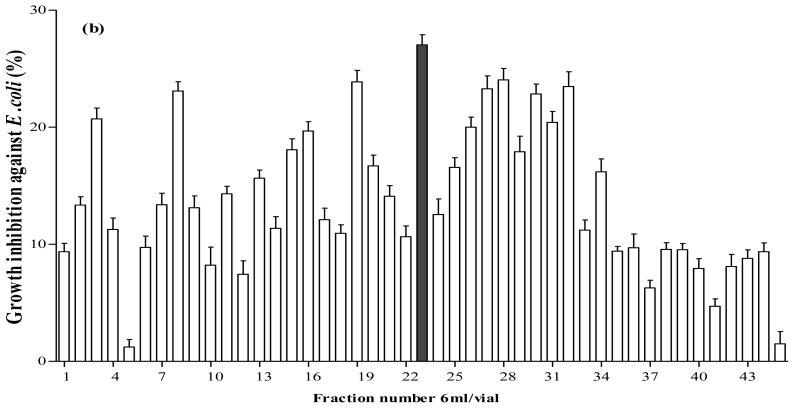
Fractionation of *A. lecanora* bromelain generated hydrolysate (after 7 h hydrolysis) by semi-preparative RP-HPLC. (**a**) Chromatogram of *A. lecanora* hydrolysate; and (**b**) Growth inhibition (%) of collected fractions against *E. coli*.

**Figure 6:**
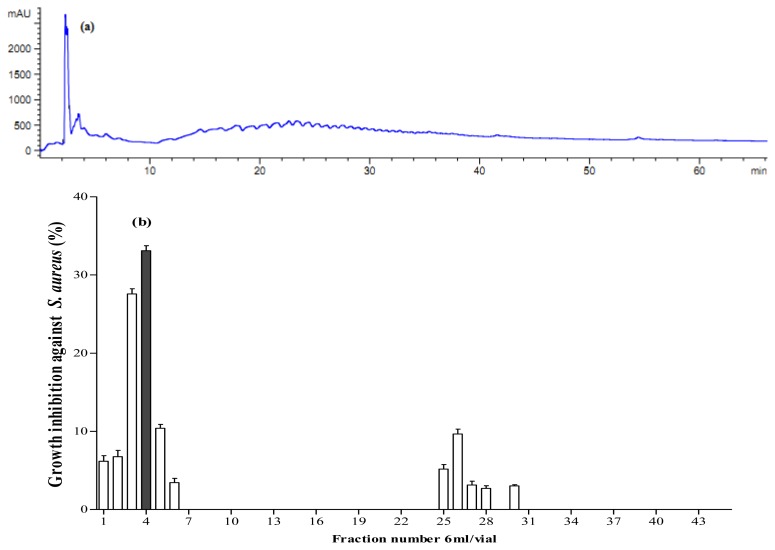
Fractionation of *A. lecanora* papain generated hydrolysate (after 8 h hydrolysis) by semi-preparative RP-HPLC. (**a**) Chromatogram of *A. lecanora* hydrolysate; and (**b**) Growth inhibition (%) of collected fractions against *S. aureus.*

The collected fractions from papain after 8 h hydrolysis and bromelain after 7 h hydrolysis effectively inhibited growth of *S. aureus* and *E. coli*, respectively, while fractions of 1 h bromelain hydrolysis effectively inhibited the growth of *Pseudomonas* sp. and *P. aerogeniosa.* All of the forty five fractions were collected after RP-HPLC fractionation and freeze dried prior to antibacterial activity determination. Among the bromelain fractions, fractions 23 and 10 inhibited the growth of *Pseudomonas* sp. and *P. aerogenios* by 24% and 25.5%, respectively (Figure 4). In addition, fraction 23, which was obtained from bromelain proteolysis after 7 h, inhibited the growth of *E. coli* by 27.1% (Figure 5). Furthermore, papain fraction 4, after 8 h hydrolysis had inhibitory activity of 33.1% against *S. aureus* (Figure 6).

**Figure 7:**
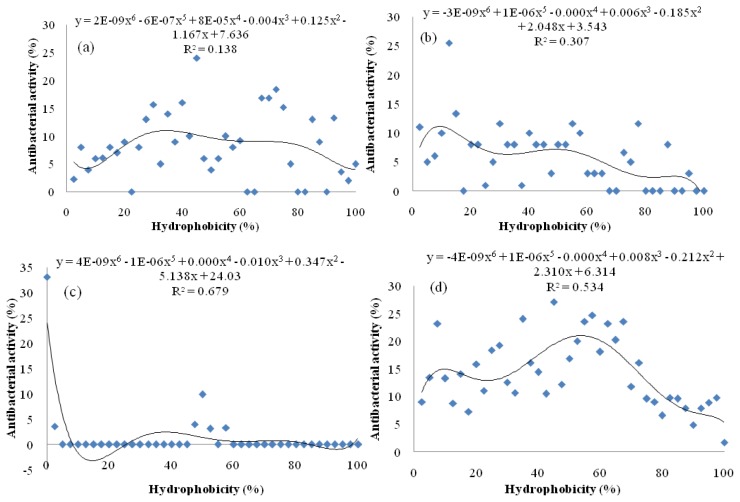
Effect of peptide hydrophobicity (%) on its antibacterial activity (%). Function of hydrophobicity (%) *versus* antimicrobial activity: (**a**) *Pseudomonas* sp.; (**b**) *P. aerogeniosa*; (**c**) *S. aureus*; and (**d**) *E. coli*.

Results revealed that peptides with mild hydrophobicity had strong antibacterial activity against *Pseudomonas* sp. and *E. coli* (Figure 7a,d) but peptides with low hydrophobicity had strong effect on *Pseudomonas aeruginosa* and *S. aureus* (Figure 7b,c). These findings demonstrated that antibacterial activity of *A. lecanora* protein hydrolysates were not correlated only to the size, molecular weight and degree of hydrolysis, but also hydrophobicity of peptides that could be attributed by the presence of hydrophobic amino acids such as leucine, isoleucine and phenylalanine [40].

## 3. Experimental Section

### 3.1. Materials and Chemicals

Alcalase and flavourzyme were obtained from Novoenzyme (Denmark). Pepsin and o-phtaldialdehyde (OPA) were purchased from Sigma-Aldrich (Munich, Germany). Papain and bromelain were obtained from Acros Organics Co. (St. Louis, MO, USA). Trypsin was purchased from Fisher Scientific (Georgia, US). Trifluoroacetic acid and all solvents used in this research were HPLC grade and obtained from Acros Organics Co. (St. Louis, MO, USA).

### 3.2. Samples

The fresh samples (*Actinopyga lecanora*) were obtained from a local supplier in Malaysia. The samples were kept in ice during transportation to the laboratory. After arrival, the internal organs were removed, and samples were washed, packed in plastic bags and kept at −80 °C, until used. Samples were freeze dried and ground with a waring blender, sieved and kept at −80 °C for further use.

### 3.3. Proximate Analysis

The chemical composition of freeze dried *A. lecanora* was determined. Total lipid content was determined based on the AOAC official method 948.15 by soxhlet extraction method for 6 h. Moisture content was quantified by the oven-drying method at 105 °C by drying the sample to a constant weight based on the AOAC 952.08 method. Ash content was determined by incineration in a muffle furnace at 550 °C for 24 h according to the AOAC method 938.08. Crude protein using Kjeldahl method was quantified according to the AOAC method 981.10 [41]. Carbohydrate was calculated by difference [12].

### 3.4. Preparation of A. lecanora Protein Hydrolysate

Powdered and freeze dried sample (10 g) was dialyzed in a 12–14 kDa molecular mass cut-off dialysis tube against deionized water followed by appropriate buffer solutions, as stated in Table 2, for 24 h. After dialysis, the sample was well mixed with enzyme and respective buffer in a test tube with the ratio of enzyme/substrate at 1/100 (*w*/*w*). Hydrolysis was performed for 24 h in a water bath shaker at the optimum condition of each enzyme (Table 2). During the hydrolysis process, the enzyme was re-added twice after 5 and 10 h reaction at the same concentration. Samples were withdrawn at one-hour intervals, starting from time 0 (before adding enzyme) to the tenth hour and one sample at the end of hydrolysis (24 h). The reaction was terminated in a boiling-water bath for 15 min to inactive the enzyme. Each protein hydrolysate was centrifuged at 10,000× *g*, 4 °C for 20 min. The supernatant was collected, filtered through 0.20 μM pore size membrane and stored at −80 °C for further analysis.

### 3.5. Determination of the Degree of Hydrolysis

The degree of hydrolysis (DH) was determined using the o-phthaldialdehyde (OPA) spectroscopic method [42] with some modifications as described by Zarei *et al.* (2012) [35]. The fresh OPA solution was prepared daily by mixing 25 mL of 100 mM sodium tetraborate, 2.5 mL 20% (*w*/*w*) sodium dodecyl sulfate (SDS), 0.16 g of OPA reagent dissolved in 4 mL ethanol (96%) and 400 μL of β-mercaptoethanol. The final volume was adjusted to 200 mL with deionized water. Assay was performed by mixing 36 μL of each protein hydrolysate with 270 μL OPA solutions in a well of 96-well plate. The mixture was incubated at room temperature for two minutes, followed by measurement of absorbance at 340 nm [43], using a 96-well plate reader (Power Wave, X340, BioTek instruments, INC. Winooski, VT, USA).

### 3.6. Cultivation and Bacterial Inoculum Perpetration

Antibacterial activity of peptides was evaluated against both Gram negative (*Escherichia coli* (ATCC 10536), *Pseudomonas aeruginosa* (ATCC 10145) and *Pseudomonas* sp.) and Gram positive (*Bacillus subtilis* (ATCC 11774) and *Staphylococcus aureus* (ATCC 25923)). Selected bacteria were cultivated aerobically at 37 °C overnight for 18 h in sterile tripton soy broth (TSB). The prepared cultures were re-cultivated for acquiring maximum growth under the same conditions, by transferring 0.5 mL of the culture into fresh medium (TSB). Bacterial inocula were prepared for antibacterial assay from the mid-logarithmic phase of their growth culture. The optical density cultures were measured at 630 nm and adjusted to around 0.5 by addition of the TSB (OD_630_ = 0.5) which contains approximately 10^8^ colony-forming units per milliLiter (cfu/mL) [44].

### 3.7. Determination of Antibacterial Activity

Antibacterial activity of bioactive peptides was evaluated following the method described by Mandar *et al.* (2011) [44] with some modifications. Each sample was prepared by mixing the bacterial inoculum (10 μL) containing 10^6^ (cfu/mL), TSB medium (90 μL) and protein hydrolysate (90 μL) into each well of 96-well plate. Wells without peptide were considered as a control and containing medium, bacterial culture and 50 mM appropriate buffer for each hydrolysate. The plates were incubated at 37 °C overnight with shaking (90 rpm) and bacterial growth was monitored by measuring the absorbance of wells at 630 nm using 96-well plate readers (Power Wave, X340, Bio Tek instruments, INC.). The percentage of inhibition was calculated as [(OD_control_ − OD_sample_)/OD_control_)] × 100. All experiments were performed in six replicates for each sample.

### 3.8. Fractionation of *A. lecanora* Peptide

Protein hydrolysate with antibacterial property was fractionated by semi-preparative RP-HPLC. Each active hydrolysate was filtered through 0.45 and 0.20 μM pore size membranes (Sartorius Stedim) before being loaded into a preparative HPLC system (Agilent Technologies 1200 series), coupled with a MWD detector and fraction collector. Separation was performed in a zorbax 300 SB-C18 column (5 μm, 9.4 mm × 250 mm, Agilent Technologies, USA) at a flow rate of 4 mL/min. The sample was eluted using two mobile phases; deionized water containing 0.1% trifluoroacetic acid (phase A) and acetonitrile containing 0.1% trifluoroacetic acid (phase B). Sample injection volume and concentration were 500 μL and 0.5 mg of peptide per milliliter, respectively. Elution was carried out at room temperature according to the following process: 0–5 min, 100% eluent A; 5–60 min, 0%–100% eluent B. Peptides were detected at 205 nm. The column was conditioned between two successive runs for 60 min using acetonitrile containing 0.1% trifluoroacetic acid. All fractions collected were freeze dried and dissolved in deionized water for antibacterial activity assay.

## 4. Statistical Analysis

All results were shown as means of three replicates. The one-way ANOVA was used for data analysis followed by Tukey’s test to identify significant differences between treatments (*p* < 0.05) with Minitab version 14 (Minitab Inc., State College, PA, USA).

## 5. Conclusion

This study demonstrated that due to its relatively high protein content, *A. lecanora* can be used as a raw material for the generation of bioactive peptides. Therefore, *A. lecanora* was hydrolyzed by different type of proteases, namely papain, alcalase, bromelain, pepsin, flavourzyme and trypsin, in order to produce antibacterial peptides. The results revealed that the antibacterial activity and characteristics of peptides produced during the hydrolysis were strongly related to the type of enzyme employed. Of the different proteases tested, papain and bromelain were found to be the most efficient for the production of hydrolysates with antibacterial properties against the selected pathogenic bacteria. The evaluation of the relationship between DH and antibacterial activities of papin and bromelain hydrolysates revealed meaningful correlations of four and six order functions. Based on our results, mild hydrophobic peptides showed higher antibacterial activity. Although most of the antibacterial peptides generated from *A. lecanora* are not comparable to synthetic antibacterial agents against the pathogenic bacteria, it is worthy to note that as natural products they generally regarded as safer and devoid of side effects. Thus, *A. lecanora* hydrolysates can be considered as a suitable natural antibacterial source alternative to chemical food preservatives for the prevention of bacteria growth in food systems and as preservatives to improve shelf life, in addition to their food safety and nutritional value. The need for further research to identify peptides that are responsible for this biological activity and its optimized production for application in food systems is deemed necessary.

## Figures and Tables

**Table 1:** Proximate compositions of fresh *A. lecanora* (wet weight basis %) in comparison to other species.

Components	Amount (%)	

	a*A. lecanora*	Other species
Moisture	87.96 ± 0.49	82–92.6
Protein	7.03 ± 0.16	2.5–13.8
Fat	0.65 ± 0.08	0.l–0.9
Ash	2.93 ± 0.07	l.5–4.3
Carbohydrate	1.43 ± 0.09	0–2.2

a Mean ± standard deviation of triplicates.

**Table 2:** Optimum conditions for enzymatic hydrolysis of *A. lecanora* by different proteolytic enzymes [35].

Enzyme	Buffer (50 mM)	pH	Temperature (°C )	Agitation rate (rpm)
Papain	Phosphat	7	60	150
Pepsin	KCl-HCl	1.5	37	150
Trypsin	Borate	8	37	150
Alcalase	Borate	8	55	150
Bromelain	Acetate	5.5	55	150
Flavourzyme	Phosphat	6.5	55	150
